# Supplementary material for: Synergy-based feedforward with minimal feedback control predicts walking over multiple cycles
Source: Front Bioeng Biotechnol. 2026 Aug 3;14:1824839. doi: 10.3389/fbioe.2026.1824839 (PMC13478117; doi:10.3389/fbioe.2026.1824839)
Supplement: Supplementary file 1 [file DataSheet1.pdf]

Supplementary Material

1 Model Parameters and Optimization Settings

Supplementary Table 1. Maximum allowable errors in Ground Contact Personalization (GCP) cost terms. GCP is formulated as a three-stage optimization, gradually changing the focus of tracking from vertical ground reaction forces to first three-dimensional ground reaction forces and then all ground reactions.

Cost Term	Stage 1	Stage 2	Stage 3
**Marker position tracking (mm)**	2	2	2
**Rotation tracking (rad)**	0.0087	0.0087	0.0087
**Vertical ground reaction tracking (N)**	5	10	10
**Horizontal ground reaction tracking (N)**	20	2	2
**Ground reaction moment tracking (Nm)**	10	10	0.5
**Spring constant neighbor deviation (N/m)**	100	100	100

Supplementary Table 2. Maximum allowable errors in torque-driven Tracking Optimization cost terms.

**Cost Term**	**Maximum Allowable Error**
**Marker position tracking (mm)**	5
**Marker velocity tracking (mm/s)**	50
**Inverse dynamics load tracking (Nm)**	10
**Generalized coordinate tracking (mm) (pelvis translation)**	20
**Generalized coordinate tracking (rad) (most rotational coordinates)**	0.1745
**Generalized coordinate tracking (rad) (subtalar, ankle)**	0.0873
**Generalized coordinate tracking (rad) (lumbar extension)**	0.6981
**Generalized coordinate tracking (rad) (upper body)**	0.3491
**Generalized speed tracking (m/s) (pelvis translation)**	0.2
**Generalized speed tracking (rad/s) (most rotational coordinates)**	1.745
**Generalized speed tracking (rad/s) (subtalar, ankle)**	0.8727
**Generalized speed tracking (rad/s) (upper body)**	3.491
**Generalized acceleration tracking (m/s^2) (pelvis translation)**	2
**Generalized acceleration tracking (rad/s^2) (most rotational coordinates)**	17.453
**Generalized acceleration tracking (rad/s^2) (subtalar, ankle)**	8.727
**Generalized acceleration tracking (rad/s^2) (upper body)**	34.907
**External force tracking (N)**	40
**External moment tracking (Nm)**	10

Supplementary Table 3. Maximum errors in torque-driven Tracking Optimization constraint terms.

Constraint Term	Maximum Error
**Generalized coordinate periodicity (rad)**	0.0873
**Generalized speed periodicity (rad)**	0.873
**Initial external force deviation (N)**	1
**Final external force deviation (N)**	1
**Initial external moment deviation (Nm)**	10
**Final external moment deviation (Nm)**	10
**Kinetic consistency (Nm)**	0.1
**Root segment residual load (N) (pelvis forces)**	1
**Root segment residual load (Nm) (pelvis moments)**	0.1

Supplementary Table 4. Maximum allowable errors and error centers in Muscle-Tendon Personalization cost terms.

Cost Term	Maximum Allowable Error	Error Center
**Inverse dynamics moment tracking (Nm) (including extrapolated muscles)**	0.25	0
**Inverse dynamics moment tracking (Nm) (excluding extrapolated muscles)**	1	0
**Extrapolated muscle activation minimization**	0.025	0
**Residual muscle activation minimization**	0.02	0
**Activation time constant regularization**	0.02	0.015
**Activation nonlinearity constant regularization**	0.01	0
**Optimal muscle fiber length regularization**	0.1	0
**Tendon slack length regularization**	0.1	0
**EMG scale factor regularization**	0.2	0.3
**Normalized muscle fiber length regularization**	0.01	0
**Passive muscle force minimization**	10	0
**Grouped normalized fiber length similarity**	0.05	0
**Grouped EMG scale factor similarity**	0.1	0
**Grouped electromechanical delay similarity**	0.2	0
**Muscle excitation minimization**	0.35	0.5

Supplementary Table 5. Maximum allowable errors in Neural Control Personalization cost terms.

Cost Term	Maximum Allowable Error
**Inverse dynamics moment tracking (Nm)**	1.5
**Muscle activation tracking**	0.05
**Grouped muscle activation similarity**	0.1

Supplementary Table 6. Maximum allowable errors in synergy-driven Tracking Optimization cost terms.

Cost Terms	Maximum Allowable Error
**Body orientation tracking (rad)**	0.0873
**Body angular velocity tracking (rad/s)**	0.873
**Generalized coordinate tracking (rad) (lower body)**	0.0873
**Generalized coordinate tracking (rad) (upper body)**	0.3491
**Generalized speed tracking (rad/s) (lower body)**	0.873
**Generalized speed tracking (rad/s) (upper body)**	3.491

Supplementary Table 7. Maximum errors in synergy-driven Tracking Optimization constraint terms.

Constraint Term	Maximum Error
**Feedforward command deviation**	0.01
**Generalized coordinate periodicity (rad) (lower body)**	0.0458
**Generalized coordinate periodicity (m) (pelvis translation)**	0.0188
**Generalized speed periodicity (rad/s) (lower body)**	0.4583
**Generalized speed periodicity (m/s) (pelvis translation)**	0.1877
**Initial generalized coordinate deviation (rad) (lower body)**	0.0349
**Initial generalized coordinate deviation (m) (pelvis translation)**	0.02
**Initial generalized speed deviation (rad/s) (lower body)**	0.349
**Initial generalized speed deviation (m/s) (pelvis translation)**	0.2
**Initial external force deviation (N)**	10
**Initial external moment deviation (Nm)**	2
**External force periodicity (N)**	14.721
**External moment periodicity (Nm)**	4.113
**Kinetic consistency (Nm)**	1
**Root segment residual load (N) (pelvis forces)**	1
**Root segment residual load (Nm) (pelvis moments)**	0.1

2 Computational Performance

Simulation steps were performed using a Windows 11 PC using an AMD processor with 16 cores at a base clock speed of 4.30 GHz.

Supplementary Table 8. Runtimes for NMSM Pipeline tools. For Tracking Optimization, an average and standard deviation are reported for testing trials, using 100% feedforward controls for the synergy-driven case.

Tool	Runtime (hours)
**Joint Model Personalization**	0.87
**Ground Contact Personalization**	1.32
**Torque-driven Tracking Optimization**	4.00 ± 0.53
**Muscle-Tendon Personalization**	0.68
**Neural Control Personalization**	6.81
**Synergy-driven Tracking Optimization**	**2.58 ± 0.04**

3 Models without Feedback

Supplementary Table 9. Joint angle tracking errors for simulations without feedback controls. As two of the three testing cycles failed in the 75% feedforward case, results are included for only the successful simulation.

FF contribution	75%	100%
**Hip flex. (R)**	7.71	2.22 ± 0.85
**Hip add. (R)**	2.53	2.06 ± 0.18
**Knee angle (R)**	12.1	3.82 ± 0.95
**Ankle angle (R)**	4.04	1.40 ± 0.16
**Subtalar (R)**	4.78	4.69 ± 0.37
**Hip flex. (L)**	3.72	3.27 ± 0.42
**Hip add. (L)**	3.40	3.08 ± 0.83
**Knee angle (L)**	7.39	4.81 ± 0.61
**Ankle angle (L)**	2.91	1.87 ± 0.17
**Subtalar (L)**	7.66	7.54 ± 0.84

Supplementary Table 10. Joint moment tracking errors for simulations without feedback controls. As two of the three testing cycles failed in the 75% feedforward case, results are included for only the successful simulation.

FF contribution	75%	100%
Hip flex. (R)	3.45	4.80 ± 1.55
Hip add. (R)	8.74	3.37 ± 0.45
Knee angle (R)	11.7	5.08 ± 1.86
Ankle angle (R)	11.3	9.32 ± 2.24
Subtalar (R)	1.09	1.24 ± 0.27
Hip flex. (L)	4.56	4.46 ± 1.84
Hip add. (L)	14.6	5.96 ± 0.57
Knee angle (L)	10.3	7.08 ± 1.89
Ankle angle (L)	4.54	9.55 ± 2.14
Subtalar (L)	1.49	1.82 ± 0.26

Supplementary Table 11. Ground reaction tracking errors for simulations without feedback controls. As two of the three testing cycles failed in the 75% feedforward case, results are included for only the successful simulation.

FF contribution	75%	100%
Ant. F (R)	12.1	15.05 ± 3.30
Vert. F (R)	66.9	67.45 ± 15.05
Lat. F (R)	5.02	8.76 ± 2.60
Front. M (R)	1.38	1.54 ± 0.23
Trans. M (R)	1.53	1.78 ± 0.22
Sag. M (R)	11.7	7.91 ± 2.57
Ant. F (L)	15.6	22.60 ± 3.69
Vert. F (L)	71.7	63.06 ± 9.88
Lat. F (L)	18.7	10.04 ± 3.38
Front. M (L)	2.07	2.34 ± 0.17
Trans. M (L)	2.27	1.31 ± 0.14
Sag. M (L)	7.28	3.69 ± 1.68

4 Individual Cycle Results

Supplementary Table 12. Joint angle tracking errors for the first testing cycle.

FF contribution	0%	25%	50%	75%	100%	125%
Hip flex. (R)	15.46	13.62	9.92	7.92	4.87	1.33
Hip add. (R)	13.94	9.40	5.16	2.12	1.93	2.20
Knee angle (R)	19.78	17.78	15.71	12.19	5.85	6.03
Ankle angle (R)	12.96	8.65	8.84	3.55	1.54	3.97
Subtalar (R)	15.04	6.04	11.15	4.74	5.60	3.84
Hip flex. (L)	13.87	9.31	5.66	3.29	3.45	6.35
Hip add. (L)	14.77	10.05	5.79	2.89	2.13	5.40
Knee angle (L)	14.39	13.01	12.29	7.56	5.85	9.99
Ankle angle (L)	8.33	6.16	9.66	3.77	1.56	2.61
Subtalar (L)	15.69	7.42	19.50	8.43	7.52	6.92

Supplementary Table 13. Joint moment tracking errors for the first testing cycle.

FF contribution	0%	25%	50%	75%	100%	125%
Hip flex. (R)	17.51	12.61	7.54	3.72	4.26	5.97
Hip add. (R)	34.86	26.56	16.05	7.66	2.76	7.20
Knee angle (R)	11.43	13.69	14.65	11.72	4.48	10.25
Ankle angle (R)	29.42	23.16	17.25	11.03	9.01	20.63
Subtalar (R)	2.88	1.16	2.55	1.31	1.06	2.67
Hip flex. (L)	18.93	14.14	9.30	4.14	2.14	4.60
Hip add. (L)	41.21	34.04	22.71	12.09	4.99	7.46
Knee angle (L)	10.05	11.01	12.09	10.46	9.77	9.00
Ankle angle (L)	18.45	16.66	11.11	4.16	7.53	12.83
Subtalar (L)	2.59	2.38	4.08	1.85	2.14	2.06

Supplementary Table 14. Ground reaction tracking errors for the first testing cycle.

FF contribution	0%	25%	50%	75%	100%	125%
Ant. F (R)	36.93	29.20	17.48	12.99	10.43	15.19
Vert. F (R)	167.76	115.31	73.31	62.55	68.90	85.63
Lat. F (R)	27.81	20.36	10.08	6.07	11.23	10.24
Front. M (R)	5.43	2.03	1.82	1.54	1.46	2.76
Trans. M (R)	4.67	3.56	1.97	1.80	1.78	3.22
Sag. M (R)	29.10	24.01	18.46	11.31	5.47	18.41
Ant. F (L)	44.35	32.75	17.11	15.22	26.47	18.63
Vert. F (L)	160.57	134.07	76.24	63.21	68.64	81.36
Lat. F (L)	27.71	22.70	18.73	19.21	12.40	14.26
Front. M (L)	4.52	3.24	4.09	1.72	2.19	2.69
Trans. M (L)	4.32	1.67	1.84	2.22	0.93	2.53
Sag. M (L)	21.17	20.44	9.68	6.80	9.95	13.13

Supplementary Table 15. Joint angle tracking errors for the second testing cycle.

**FF contribution**	**0%**	**25%**	**50%**	**75%**	**100%**	**125%**
**Hip flex. (R)**	17.32	12.22	8.01	7.06	2.44	3.51
**Hip add. (R)**	8.79	7.15	4.52	1.30	2.02	2.97
**Knee angle (R)**	16.58	18.17	15.74	10.36	4.34	6.10
**Ankle angle (R)**	12.36	9.48	10.82	3.27	1.20	2.26
**Subtalar (R)**	15.65	5.24	14.71	3.62	5.46	3.64
**Hip flex. (L)**	17.46	9.58	6.07	3.50	4.48	6.23
**Hip add. (L)**	10.24	8.50	5.58	3.62	3.63	5.24
**Knee angle (L)**	16.17	12.64	11.44	6.83	5.85	9.54
**Ankle angle (L)**	9.72	8.22	11.72	4.25	2.29	2.49
**Subtalar (L)**	13.91	8.93	24.08	10.23	8.55	6.77

Supplementary Table 16. Joint moment tracking errors for the second testing cycle.

**FF contribution**	**0%**	**25%**	**50%**	**75%**	**100%**	**125%**
**Hip flex. (R)**	13.09	8.53	4.22	4.76	6.99	8.26
**Hip add. (R)**	33.14	25.09	14.03	6.42	3.48	8.57
**Knee angle (R)**	16.17	18.59	20.79	15.05	8.08	7.05
**Ankle angle (R)**	24.50	19.02	12.94	6.55	12.21	24.29
**Subtalar (R)**	1.89	0.82	4.21	1.52	1.34	3.03
**Hip flex. (L)**	20.43	15.70	9.32	5.07	5.04	7.31
**Hip add. (L)**	39.43	33.37	20.36	11.89	5.74	5.56
**Knee angle (L)**	7.45	8.00	9.05	7.82	7.61	8.05
**Ankle angle (L)**	27.32	24.76	20.08	11.71	9.00	10.89
**Subtalar (L)**	2.91	3.15	4.62	1.99	1.76	1.73

Supplementary Table 17. Ground reaction tracking errors for the second testing cycle.

**FF contribution**	**0%**	**25%**	**50%**	**75%**	**100%**	**125%**
**Ant. F (R)**	36.92	23.86	16.92	12.01	15.57	28.18
**Vert. F (R)**	137.00	129.41	48.15	53.35	77.37	78.60
**Lat. F (R)**	29.77	25.84	19.20	11.52	10.49	10.51
**Front. M (R)**	4.76	2.81	3.82	1.81	2.00	3.11
**Trans. M (R)**	4.81	3.29	2.01	1.72	2.14	3.96
**Sag. M (R)**	24.18	21.04	13.17	7.07	10.80	23.91
**Ant. F (L)**	42.13	26.31	10.58	13.82	32.33	28.89
**Vert. F (L)**	146.52	84.01	67.46	53.96	69.67	77.39
**Lat. F (L)**	28.00	22.84	19.38	18.90	11.00	8.13
**Front. M (L)**	6.91	4.37	3.74	1.51	2.23	2.33
**Trans. M (L)**	3.88	2.20	1.65	2.79	1.35	1.79
**Sag. M (L)**	27.37	26.71	14.96	9.73	7.39	9.65

Supplementary Table 18. Joint angle tracking errors for the third testing cycle.

FF contribution	0%	25%	50%	75%	100%	125%
**Hip flex. (R)**	16.19	12.61	7.51	6.34	1.53	2.32
**Hip add. (R)**	14.09	9.98	5.27	2.24	2.12	2.32
**Knee angle (R)**	22.52	17.97	14.66	10.75	4.68	6.98
**Ankle angle (R)**	11.98	7.68	9.07	3.81	1.56	2.33
**Subtalar (R)**	12.74	4.70	11.00	3.73	4.78	3.26
**Hip flex. (L)**	15.81	10.96	7.45	6.04	4.77	5.28
**Hip add. (L)**	15.25	11.04	6.35	2.20	1.76	3.45
**Knee angle (L)**	16.12	14.70	14.75	9.03	6.49	7.69
**Ankle angle (L)**	11.06	8.20	11.42	5.40	2.38	2.70
**Subtalar (L)**	20.55	8.87	21.55	7.88	7.03	3.83

Supplementary Table 19. Joint moment tracking errors for the third testing cycle.

FF contribution	0%	25%	50%	75%	100%	125%
**Hip flex. (R)**	16.86	12.06	6.70	3.60	4.22	5.63
**Hip add. (R)**	33.67	25.49	14.46	7.48	3.50	9.19
**Knee angle (R)**	12.19	14.21	15.53	11.99	4.37	8.03
**Ankle angle (R)**	30.21	22.73	16.83	8.22	6.19	18.75
**Subtalar (R)**	3.79	2.33	2.35	2.19	1.94	2.53
**Hip flex. (L)**	21.29	16.92	11.36	7.77	6.45	11.00
**Hip add. (L)**	40.26	33.74	22.61	11.37	5.01	11.04
**Knee angle (L)**	7.04	6.61	6.98	5.19	4.97	5.87
**Ankle angle (L)**	32.55	29.35	24.51	18.38	11.96	11.05
**Subtalar (L)**	4.23	4.06	3.73	2.54	1.30	1.87

Supplementary Table 20. Ground reaction tracking errors for the third testing cycle.

FF contribution	0%	25%	50%	75%	100%	125%
**Ant. F (R)**	39.97	32.30	22.82	15.70	14.23	24.87
**Vert. F (R)**	207.17	137.65	67.00	57.02	31.18	46.37
**Lat. F (R)**	25.05	18.15	13.36	15.62	6.80	7.25
**Front. M (R)**	5.44	3.22	2.20	2.11	1.16	2.46
**Trans. M (R)**	4.24	2.77	1.76	2.13	1.76	2.91
**Sag. M (R)**	27.00	24.86	17.04	9.52	6.37	19.26
**Ant. F (L)**	44.77	28.91	9.91	19.79	19.13	22.59
**Vert. F (L)**	183.34	122.69	52.95	67.90	43.98	46.40
**Lat. F (L)**	38.26	22.07	17.43	19.49	12.10	10.69
**Front. M (L)**	5.03	4.08	2.89	3.38	2.68	1.78
**Trans. M (L)**	4.40	2.27	1.40	3.84	1.09	2.59
**Sag. M (L)**	38.05	32.54	21.41	18.68	10.96	11.64
